# Great Things

**Published:** 1882-11

**Authors:** 


### GREAT THINGS.

Fortress Monroe is the largest single fortification in the world. It has already cost the Government over $3,000,000. The water battery is considered one of the finest military works in the world.

The Chinese wall is the largest wall in the world. It was built by the first Emperor of the Tain dynasty, about 220 B. C., as a protection against Tartars. It traverses the northern boundary of China, and is carried over the highest hills, through the deepest valleys, across rivers and every other natural obstacle. Its length is 1,250 miles. Including a parapet of 5 feet, the total height of the wall is 20 feet, thickness at the base 25 feet, and at the top 15 feet. Towers or bastions occur at intervals of about 100 yards.

The largest bell in the world is the great bell of Moscow, at the foot of the Kremlin. Its circumference at the bottom is nearly 68 feet, and its height more than 21 feet. In its stoutest part it is 23 inches thick, and its weight has been computed to be 443,772 pounds. It has never been hung, and was probably cast on the spot where it now stands. A piece of the bell is broken off. The fracture is supposed to have been occasioned by water having been thrown upon it when heated by the building erected over it being on fire.

Among the most remarkable natural echoes is that of Eagle's Nest, on the banks of Killarney, in Ireland, which repeats a bugle call until it seems to be sounded from a hundred instruments ; and that on the banks of the Nahe, between Bingen and Coblentz, which repeats a sound seventeen times. The most remarkable artificial echo known is that in the Castle of Simonetta, about two miles from Milan. It is occasioned by the existence of two parallel walls of considerable length. It repeats the report of a pistol sixty times.

The largest library is the Bibliotheque Nationale in Paris, founded by Louis XIV. It contains 1,400,000 volumes, 300,000 pamphlets, 175,000 manuscripts, 300,000 maps and charts, and 150,000 coins and medals. The collection of engravings exceeds 1,300,000, contained in some 10,000 volumes. The building which contains these treasures is situated on the Rue Richelieu. Its length is 540 feet, its breadth 130 feet. The largest library in New York, in respect of separate works, is the Astor. About 190,000 volumes are on its shelves.

The largest cathedral in the world is St. Peter's, in Rome. From the laying of its foundation in 1450 until its dedication, one hundred and seventy years were consumed in its erection ; and if we include the work done under Pius VI., three and a half centuries pas ed before it was complete, during which time forty-three Popes reigned. The dimensions of the church are : Length of interior, 613½ feet ; of transom from wall to wall, 446½ feet ; height of nave, 152½ feet ; of side aisles, 47 feet ; width of nave, 77-89 feet ; of side aisles, 33¾ feet ; circumference of pillars which support the dome, 253 feet. The height of the dome from the pavement to the base of the lantern is 405 feet—to the top of the cross, 448 feet. The dome is encircled and strengthed by six bands of iron. A stairway leads to the roof, broad and easy enough to allow a horse and team to ascend. The annual cost of keeping the church in repair is 30,000 scudi.

